# Child Welfare Work in Bradford

**Published:** 1916-04-22

**Authors:** 


### CHILD WELFARE WORK IN BRADFORD.

### Hospital and Municipal Work Compared.

When referring, in a recent article,* to Dr. H. G. Campbell's criticism of the Bradford Municipal Child Welfare and Maternity Scheme, we dealt with the suggestion that this kind of work might have been expected to have a greater effect upon the infant mortality rate of a large city; we now endeavour to examine in what way the two forms of public service cover the same ground, and in this way raise questions of needless expenditure of public money. Let us first review the work of the medical voluntary charities in Bradford so far as it relates to children.

### The Hospitals.

In addition to the treatment given under the direction of the medical staff to the children patients at the Bradfield Royal Infirmary, what else is done for them in the way of after-care, in order that the good they have derived through the hospital shall have a lasting beneficial effect? At this hospital, where in 1915 181 was the average number of beds occupied daily, and the out-patients and casualty cases made an aggregate of 55,000 attendances, there is no almoner's department. There is, however, a Samaritan Society, and, in addition, suitable cases are referred to the Charity Organisation Society. The Samaritan Society is not a large one; its total income last year was £176, and the funds of late years have been steadily decreasing. The number of patients helped in 1915 was 159; and assistance took the form of food, coal, clothing, part cost of surgical appliances, and railway fares to the hospital. The benefits of the society were not given exclusively to infirmary patients. One case quoted in the report as typical of the work done is that of a boy suffering from hip-joint disease, who is helped with his weekly payments at a Cripples' Home, where he is "in the open-air ward, and deriving much benefit."

At the Royal Infirmary last year 364 children were treated in the wards, and 418 as out-patients. There is a Convalescent Home for children at Morecambe managed in connection with the infirmary, but the patients received do not all come from there—in fact, only thirty-four of the 294 admissions in 1914 had been hospital patients. The period of residence is three weeks. It is not clear from the report of the home if all the patients come from the City of Bradford or from various localities.

In surveying what is done for children in Bradford the work of the Children's Hospital must not be overlooked. Here, in 1914, 704 patients passed through the wards, 285 of whom were operation cases. The out-patient attendances numbered 17,124. After-care is in the hands of a ladies' committee, who in 1914 visited 372 children at their homes after they had been discharged. In many cases milk and other nourishing food was provided for a few weeks through a Convalescent Fund.

In summing up, it will be seen that by far the greater part of the work done for children by the Bradford hospitals is in affording medical and surgical attention, and that any attempt at general child welfare work, or after-care work on a large scale, has not been made by the voluntary medical charities. In short, with the exception of the features we have quoted, it is clear that the work of the hospitals is in the main curative; but in saying this there is no intention to belittle the splendid services to the sick poor rendered by the two institutions referred to, the only object being to get at facts by which to examine Dr. Campbell's criticism.

### The Municipal Scheme.

The Bradford Municipal Scheme is very comprehensive in character. A description of the work may be conveniently divided under five heads, as follows :—

1. Ante-Natal Clinic and Maternity Home.—In this department the health of the mother before the birth of her child receives careful attention. The accommodation includes a maternity home with nine beds, a labour ward, and an operating-theatre. A fully qualified maternity nurse is supplied for extern cases at an inclusive fee of 10s.; if the case is one where it is necessary to call a doctor, his fees are paid by the Corporation. Patients entering the home pay a fee of 10s., and 1s. a day during residence.

2. Infants' Department (Consultative and Hospital) and Milk Depot.—This clinic consists of waiting, dressing, weighing, and receiving rooms. The staff consists of three whole-time medical women, a qualified dispenser, and staff nurses and assistants. Over 600 babies attend weekly, and a special effort is made to secure attendance of infants as soon as possible after birth, prevention rather than cure being the keynote of the department. A staff of women health visitors keeps in close touch with all the cases, and works on the principle that it is better to strengthen and elevate natural influences rather than to attempt to find a substitute for parental care. The milk depôt supplies the various departments, and, in addition, a number of public institutions and school feeding centres. Breast feeding of infants is advocated, but in an industrial centre cannot be ensured, as many of the child-bearing women work in factories. The milk preparations are sent to the homes in special bottles—one for each meal—and are fitted with a suitable easily cleaned rubber teat.

3. Meals for Expectant and Nursing Mothers.—Wholesome and suitable food is sent in heat-proof vessels by motor-vans to eight feeding centres. Precautions are taken to prevent abuse, the family income is ascertained, and the recipient pays when able towards the cost of food. It is also part of the bargain that she must attend the ante-natal clinic for advice, and when the child is born must take it to the infant consultations. Natural feeding is, of course, encouraged, but in many of the poorer homes malnutrition in the mother causes failure of the breast-milk secretion.

4. Pre-School and Post-School Clinic.—The effort here is to raise the general standard of health of school children and then to bridge the gulf between school and insurance age, or, in other words, the period of transition from childhood to youth.

5. Special Departments.—In these, medical and surgical treatment is given of diseases of the eye, ear, throat, and nose in children.

It would be impossible to make any fair deductions from the figures at the disposal of the public of the relative cost of the work for children at the hospitals and in the municipal centre. It is probable that the hospitals, not having the apparently bottomless purse of the ratepayer unrestrainedly to draw upon, work on less expensive lines than the Corporation. We can only ask in what ways the two branches of effort overlap. Hardly

* The Hospital, April 8, 1916.

at all is this the case in the ante-natal and maternity work, to some extent in respect of the medical side of the infants' department, not at all as regards feeding the child-bearing mothers, and to a large extent in the special departments for the treatment of diseases of children.

Speaking generally, the municipal undertaking holds an unchallenged field in its zealous regard to the general welfare of the child from before birth to puberty, continuous work which has only in a small measure to do with medicine and surgery.

The justification of the medical and surgical (or purely curative) municipal scheme lies no doubt in the praiseworthy desire to leave no feature of the work incomplete, but here there must be overlapping and unnecessary expenditure of public funds, and it seems reasonable to consider to what extent the splendid resources of voluntary hospitals in this respect might work side by side with the municipality.

But the hospitals cannot be said to have touched the fringe of the main scheme of child welfare in Bradford, and while, as in a recent year, in the county division in which the city is situated the infant death-rate in respect of premature birth (probably ascribable to pre-natal conditions) and congenital defects is as high as 27.8 per 1,000, there is an urgent public need for the best services of both physician and the specialised child welfare worker.

